# Bioactive Substances in Safflower Flowers and Their Applicability in Medicine and Health-Promoting Foods

**DOI:** 10.1155/2021/6657639

**Published:** 2021-05-26

**Authors:** I. Adamska, P. Biernacka

**Affiliations:** Department of Fish, Plant and Gastronomy Technology, West Pomeranian University of Technology in Szczecin, Szczecin, Poland

## Abstract

Safflower flowers (*Carthamus tinctorius*) contain many natural substances with a wide range of economic uses. The most famous dye isolated from flower petals is hydroxysafflor A (HSYA), which has antibacterial, anti-inflammatory, and antioxidant properties. This review is aimed at updating the state of knowledge about their applicability in oncology, pulmonology, cardiology, gynecology, dermatology, gastrology, immunology, and suitability in the treatment of obesity and diabetes and its consequences with information published mainly in 2018-2020. They were also effective in treating obesity and diabetes and its consequences. The issues related to the possibilities of using HSYA in the production of health-promoting food were also analyzed.

## 1. Introduction

The demand for natural compounds used in the key branches of economy is increasing in connection with the growing awareness of consumers. However, producers are forced to follow economic considerations and use cheaper equivalents of certain ingredients. For this reason, safflower (*Carthamus tinctorius*), treated as a low-cost replacement for saffron crocus (*Crocus sativus* L.) and as a rich source of bioactive substances, has been applied in numerous branches of economy.

Saffron is known for its rich chemical composition: simple and complex sugars, amino acids, proteins, lipids, cellulose, mineral compounds, and vitamins, including thiamine and riboflavin, low amounts of *α*-carotene and *β*-carotene, zeaxantin, and lycopene. However, the content and bioactivity of several glycosides in flowers (safranal, picrocrocin, crocetin, and crocins) are an indicator of the attractiveness of this plant [1–7]. These glycosides have not yet been found in safflower flowers.

The difference in the price of saffron and safflower as an industrial raw material depends on the crop yield. Commercial saffron (spice) accounts for only 8% of the total flower yield. One plant produces a maximum of 9 pistil stigmas (up to 3 flowers, each with 3 stigmas) [8]. The average weight of the flower pistil is about 2 mg, so a maximum of about 18 mg of raw material is obtained from one plant. It has been estimated that one kilogram of spice is made from 150,000 flowers [9]. In the case of safflower, the raw material is the whole flowers (petals with stamens) formed in large inflorescences (each contains 20-250 flowers [10]).

The purpose of the study is to review the current information (mainly published in 2018-2020) on the chemical composition of the substances present in the flowers and green parts of safflower (*Carthamus tinctorius*), their impact on humans, and the possibility of using them in the production of health-promoting food. For the first time, the possibilities of using hydroxysafflor A, the main bioactive component, were analyzed.

## 2. Botanical Characteristics and Cultivation Requirements

Safflower (*Carthamus tinctorius*) is classified in the Asteraceae family. Its natural area of distribution covers Asia (area of India) and Middle East. The plant has bushy habit and it reaches the height of 100–130 cm. It forms large, lanceolate leaves with serrated margins. Safflower flowers are radial and tubular and they form large inflorescences (flower heads) [10–12]. Four cultivar groups were distinguished depending on the color of the flowers before and after drying: (1) yellow blooming and red dried flowers, (2) yellow blooming and dried flowers, (3) orange blooming and dark red dried flowers, and (4) white blooming and dried flowers [10].

Safflower is resistant to wind, drought [13], and salinity [14]. It can be cultivated on low-fertility soils (Koutroubas and Papakosta (2005), after [15]), preferably in warm and not too humid climate. The largest plantations are located in India and Bengal, southern France, USA, Iran, Egypt, and China [13, 16, 17].

The quality of raw material obtained from safflower depends on the conditions prevailing in the cultivation area: air temperature and humidity, soil moisture, insolation, and soil fertility [15, 18–22]. The intensity of insolation affects the content of flavonoids: their synthesis was higher under the conditions of limited exposure of plants to sunlight [22]. The features of the cultivated plant are also important [15, 23–25]. Studies are currently conducted aiming at obtaining safflower cultivars with the highest possible content of pigments in flower petals [26].

## 3. Chemical Composition of Safflower Green Parts and Flowers

The above-ground parts exhibit high content of carbon (42.7–49.1% d.w.) and relatively low nitrogen (0.36–1.23%) [27]. They contain carthamusin A [17], *β*-daucosterol, and stigmasterol [28].

The chemical composition of flowers is interesting and rich (Table 1), with 200 substances identified thus far [29]. Flower petals contain 1.82% protein, 4.8% lipids, 11.6% crude fiber, and 10.8% ash, and their moisture content is 4.7% [30]. They also contain, among others, alkaloids, flavonoids, lignanoids, organic acids and polyacetylenes [17, 31], alkanediols, riboflavin, steroids, and quinochalcone C-glycosides [29].

Most of the pigments found in flower petals are flavonoids of the C-glucosylquinochalcone group. The best known are carthamine (also known as safflower yellow, carthamus red, or carthamine) and carthamidin (synonyms include carthamic acid). Carthamine (C_43_H_42_O_22_), red pigment, is flavonoid compound consisting of two chalkonoids. It is formed as a result of oxidation with precarthamine. It is insoluble in water and it usually constitutes 3–6% of petal composition; however, in some flower parts, the content is below 1% [30]. During the biosynthesis of carthamine flowers, color gradually changes from yellow to red [32]. The yellow pigment, carthamidin, constitutes 24–30% of compounds found in the flowers of safflower. It is a tetrahydroxyflavanone, (S)-naringenin derivative, water-soluble substance [30]. The following flavonoids have been identified: hydroxysafflor yellow A (HSYA; also known as safflomin A), 6-hydroxykaempferol 3,6-di-*O*-*β*-d-glucoside-7-*O*-*β*-d-glucuronide, 6-hydroxykaempferol 3,6,7-tri-*O*-*β*-d-glucoside, 6-hydroxykaempferol 3-*O*-*β*-rutinoside-6-*O*-*β*-d-glucoside, 6-hydroxykaempferol 3,6-di-*O*-*β*-d-glucoside, 6-hydroxyapigenin 6-O-glucoside-7-*O*-glucuronide, anhydrosafflor yellow B, kaempferol 3-*O*-*β*-rutinoside, and two compounds from other groups: guanosine and syringin [33]. In the course of further study, the following substances were also isolated: (2S)-4′,5-dihydroxyl-6,7-di-O-*β*-D-glucopyranosyl flavanone, 6-hydroxy kaempferol 3-O-*β*-D-glucoside, 6-hydroxykaempferol 3-O-*β*-D-rutinoside, 6-hydroxykaempferol 6,7-di-O-*β*-D-glucoside, and 6-hydroxyquercetin 3,6,7-tri-O-*β*-D-glucoside [34]. Other ingredients isolated from aqueous extracts of flower petals were quinochalcones (cartormin, isosafflomin C, precarthamine, safflomin B, safflomin C, safflor yellow A, safflor yellow B, and tinctormin [35]) and flavonoids (azaleatin (3,7,3′,4′-tetrahydroxy-5-methoxyflavone), saffloroside (3,7,3′,4′-tetrahydroxy-5-methoxyflavone 7-O-*β*-D-glucopyranoside), 5-O-methylluteolin [36, 37], cinaroside (5,7,3′,4′-tetrahydroxyflavone 7-*O*-*β*-D-glucopyranoside) [36], and 6-hydroxykaempferol-3-*O*-*β*-D-glucoside-7-*O*-*β*-D-glucuronide [38]).

Aqueous extract of flowers also contained isocartormin, new semiquinonechalcone C-glycoside, which is a cartormin isomer [29], while from immature, yellow petals of *C. tinctorius*-precarthamine, the yellow precursor of carthamine. Unripe flower petals contain an enzyme that converts precarthamine into carthamine [32].

There is a correlation between the content of active substances and the intensity of flower's color. Among the active substances, hydroxysafflor A has always been predominant (independently of the degree in which flowers are colored); however, flowers with more intense coloration (vivid red and bright yellow or bright orange) were characterized by higher content of HSYA, anhydrosafflor yellow B, kaempferol, quercetin, safflomin C, kaempferol-3-O-rutinoside, and 6-hydroxykaempferol-3–0-*β*-d-glucoside compared with less colored flowers [39, 40].

### 3.1. Essential Oils

Studies showed the presence of 20 [41] and 29 [42] substances included in the safflower essential oil. The highest content in the tests [41] characterized by heptacosane (34.8%), nonanoic acid (17.9%), and dec-2-en-1-ol (14.3%) and in study [42] 1-hydroxy-3-propyl-5-(4-methyl-penten)-2-methylbenzene (25.2%) and 2,5,5 trimethyl 3-n propyl, tetra hydro1-naphtol (19.8%) were predominant.

## 4. Applicability of Safflower Bioactive Substances in Medicine

**Figure 1:**
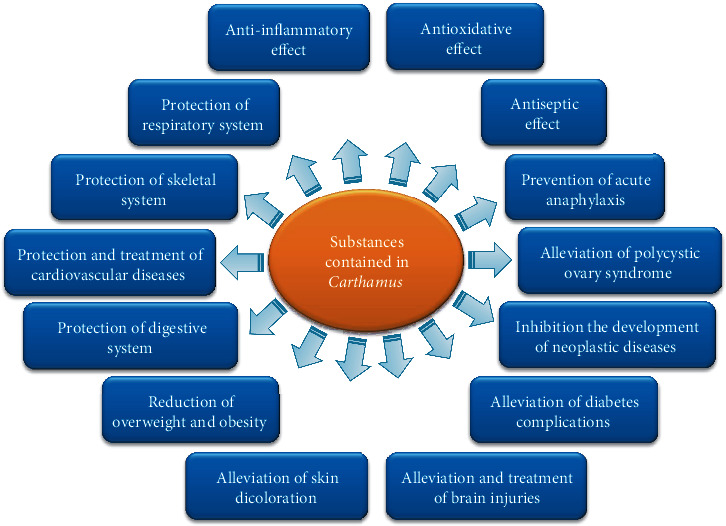
Bioactive effect of the safflower substances.

Substances isolated from safflower flowers have been used in medicine (summarized in Figure 1). Water-soluble components are particularly important in this case, especially intravenously administered quinochalcone C-glycosides [34]. Safflower is attributed with analgesic, anti-inflammatory, and antiaging properties (after [17]). Most studies have been devoted to the use of hydroxysafflor A (HSYA); however, positive impact of yellow carthamine (CY), safflor A (SA), safflower yellow (SY), hydroxysafflor B (HSYB), hydroxysafflor C (HSYC), and selected water-soluble polysaccharides on human health has been also demonstrated (summarized in Tables 2–5).

### 4.1. Antioxidative, Antiseptic, and Anti-Inflammatory Effect

Hydroxysafflor yellow A has an antioxidant effect, enabling cells to survive oxidative stress [43–50]. Antioxidant activity is also demonstrated by hydroxysafflor yellow B [51], hydroxysafflor yellow C [52], safflor A (SYA) [48], and carthamine [53]. However, safflower flower extract as well as HSYA and SYA acted in this manner only at low concentrations, whereas in higher concentrations they exhibited prooxidative effect favoring creation of reactive oxygen species in the cells [47]. The paste produced from crushed safflower leaves accelerated the healing of difficult to control wounds (Dehariya et al. (2015), after [54]). Another representative of the genus *Carthamus* (*C. oxyacantha*) also shows antibacterial and antidiarrheal effect. Substances contained in the plant limited the proliferation of *Escherichia*, *Pseudomonas*, *Salmonella*, and *Staphylococcus* [55]. Moschamine obtained from *C. tinctorius* seeds has anti-inflammatory effect: it inhibits prostaglandin E2 and nitric oxide production in macrophages [56].

### 4.2. Immune System

Products containing HSYA have enabled preventing acute anaphylaxis in mice. Anaphylaxis may appear in response to contact with allergen or drug, and it is associated with a rapid activation of mediators (i.e., tumor necrosis factor, histamine, *β*-hexosaminidase, or monocyte chemotactic protein from mast cells), which may result in death. Administration of HSYA has clearly inhibited degranulation of mast cells via impeding Ca^2+^ transport and the release of cytokines and chemokines [57].

### 4.3. Hormone and Reproductive System

HSYA injection led to reduction of cysts on mouse ovaries with polycystic ovary syndrome, and at the same time, it regulated the hormonal balance and restored normal ovulation cycle by reducing the levels of testosterone and follicle-stimulating hormone (FSH) in the blood and increasing the level of progesterone, estradiol, and luteinizing hormone [46]. Anti-Müllerian hormone level, an indicator of the ovulation process, also increased.

### 4.4. Diabetes-Related Complications

Untreated diabetes results in increased level of advanced glycation end products (AGEs) and methylglyoxal in the organism's cells, which have toxic effect on cells (apoptosis), tissues (accelerated aging), and organs (destruction). In the course of diabetes, increased content of caspase-3 is observed, which is responsible for, among others, degradation of the ICAD protein (inhibitor of caspase-activated DNase), DAFF40/CAD endonuclease inhibitor. This leads to activation of endonuclease and DNA fragmentation. Lower amounts of AGEs are formed in the organism after administration of HSYA, which impedes cell apoptosis. This compound has protective effect on the microvascular endothelium in the human brain, which reduces the range of damage caused by elevated AGE level [58].

Furthermore, hydroxysafflor A has protective effect towards pancreatic cells. High blood glucose concentration leads to oxidative damage and apoptosis of *β* cells in the pancreas, leading to its disturbed function. HSYA reduces oxidative injury of cells and results in inhibited apoptosis of *β* cells via blocking the JNK/c-Jun signaling pathway [44] and affecting the course of the PI3K-AKT signaling pathway (the phosphatidylinositol 3-kinase and AKT protein kinase pathway) [59]. HSYA reduces the parameters enabling the assessment of the level of oxidative stress (levels of catalase, glutathione peroxidase, lipid peroxidation, reactive oxygen species, and superoxide dismutase) and indicators of pancreas cell apoptosis level (content of caspase-3 and parp) [44]. HSYA administration resulted in reduced level of fasting glucose in the blood of rats and reduced insulin resistance, as well as positive effect on lipid metabolism. This decreased the total and low-density lipoprotein cholesterol and triglyceride levels, and the level of glycogen synthase and hepatic glycogen was increased [59].

Favorable impact of HSYA was also observed in the treatment of kidney fibrosis associated with diabetes in rats. This substance affected the course of TLR4/NF-*κ*B/p65 signaling pathway and produced improvement (reduction) of a series of tested parameters (including miRNA-140–5p mRNA, 24 h UP, TC, or TNF-*α* and the level of proteins: Col-IV, NF-*κ*B(p65), NLRP3, Notch2, and TLR4) and relieving the symptoms of kidney fibrosis [60].

Administration of methanol extract of safflower flowers containing phenolic compounds (i.e., acids: caffeic, catechin, catechol, chlorogenic, ellagic, protocatechuic, and vanillic) and flavonoids (i.e., hesperidin, kaempferol, narengin, quercetin, quercitrin, rosmarinic, rutin, and 7-hydroxyflavone) reduced symptoms of pancreas dysfunction in rats with diabetes. This extract exhibited high reducing force and antioxidative activity (DPPH^+^) [49].

### 4.5. Nervous System

As shown for rats, HSYA reduces the effects of brain injuries (contusions). It inhibited development of symptoms, favored the increase of superoxide dismutase and ATPase activity, increased the amount of tissue plasminogen activator, and resulted in reduced level of plasminogen-1 activator inhibitor in the blood plasma as well as malondialdehyde in the tissues adjacent to the injury [61]. Furthermore, HSYA is efficient in treatment of brain ischemic injuries [62, 63], as it inhibits activation of the pyroptosis pathway (brain cell death induced by caspase-1 resulting from nerve damage). HSYA promotes the inhibition of cell apoptosis process and enhances viability of the cells damaged as a result of oxidative stress. It is also a part of the Lex-HSYA complex which activates injury relieving factors, and this restricts the range of cerebral infarction [63]. What is more, administration of HSYA resulted in alleviation and decreasing the rate of proinflammatory and oxidative reactions in the tissues involved by ischemia-reperfusion injury of rat brain [64] and induced vasodilation of cerebral and improved their permeability [62]. The substance is also effective in the treatment of cerebrovascular injuries resulting from heat stress, as it inhibited apoptosis and autophagy of nerve stem cells and stimulated proliferation [65]. HSYA inhibited the development of Parkinson's disease symptoms by regulating the level of *α*-synuclein, which reduced synthesis of dopamine and retarded intracellular degradation of brain cells [66].

### 4.6. Skin Discolorations

HSYA has proven effective in the treatment of skin hyperpigmentation and hypopigmentation. The substance formed complexes with tyrosinase altering its activity, and as an end result, it inhibited production of melanin from tyrosine. This resulted in homogenization of the skin color [43].

### 4.7. Overweight and Obesity

HSYA reduces obesity in mice and rats. When administered orally, it resulted in a change of composition of the diet-dependent intestinal microflora. As a result of this, certain bacteria groups had markedly increased counts, while other reduced, which affected the course of digestive processes and enhanced the function of the digestive tract (i.e., the intestines), as well as systemic metabolism. The level of lysophosphatidylcholines (lyso PCs), L-carnitine, and sphingomyelin increased, whereas that of phosphatidylcholines decreased. This resulted in reduced amount of fat accumulated, restored glucose homeostasis, alleviation of insulin resistance, and reduced amount of inflammations in the organism [67]. What is more, HSYA stimulated the production of antioxidative enzymes present in the liver and adipose tissue, which reduced obesity in mice, while intercurrent administration of HSYA and safflower yellow (syn.: carthamine yellow) increased the level of mRNA of antioxidative enzymes and resulted in increased activity of superoxide dismutase (SOD) in the liver [50]. HSYA increased synthesis of hormone-sensitive lipase (HSL) and affected its activity, which inhibited adipocyte proliferation [68].

### 4.8. Skeletal System

During *in vitro* studies, safflower yellow (SY) pigment increased migration of endothelial cells of the umbilical vein and produced increase in angiogenesis and differential of bone cells via increasing levels of HIF-1*α*, VEGF, Ang-2, ALP, Runx2, and OPN-1, directly affecting the pVHL/HIF-1*α*/VEGF signaling pathway, which enables easier treatment of bone fractures [69]. Favorable impact of water-soluble polysaccharides isolated from safflower on the cells of the head of the thigh bone in rats and mice treated with steroid drugs (prolonged use of steroids results in i.a. thigh bone head osteonecrosis) was demonstrated [69–72]. Polysaccharides inhibited the activation of caspase-3 participating in the processes of cell apoptosis, which resulted in increased viability of thigh bone head cells and contained osteoblast apoptosis [70]. Following a 60-day treatment with a polysaccharide of beta-d-glucan group with (1➔3) bonds, regression of histopathological changes, reduced number of cells involved in apoptosis, and adipose cells in the bone marrow were observed, which was accompanied by reduced expression of Bax protein and caspase-3, with concomitant increase in Bcl-2 protein expression [72]. Similar effect was produced by water-soluble polysaccharide containing repeating 1,4,6-*β*-Glcp skeleton bonded with T-B-Glpc in C6 position. It enhanced bone mineral density and reduced the amount of histopathological changes, the amount of void gaps in the bone, and the apoptosis indicator for the osteocytes of the thigh bone head. It further resulted in increased level of hydroxyproline in blood serum and drop in the hexosamine blood level [71].

### 4.9. Respiratory System

HSYA produced reduction of vessel permeability, reduction of the amount of blood platelets in plasma, and reduced their aggregation in the lungs of rats with prolonged exposure to car exhaust fumes, which considerably mitigated lung injury and reduced the likelihood of other lung diseases [73]. HSYA had protective effect on the respiratory system of guinea pigs, and it alleviated the symptoms of acute lung injury, chronic obstructive pulmonary disorder, and asthma caused by ovalbumin. It inhibited the action of platelet-activation factor (PAF) in the airway epithelium and mitigated inflammatory processes by blocking synthesis of triggers: protein-1 activator, nuclear *κ*B activation factor, protein kinase C, and protein kinases activated by mitogen [74].

### 4.10. Circulatory System

In China, HSYA is recommended for the treatment of angina pectoris [75] and other circulatory disorders [76]. This substance reduced myocardial damage following ischemic/reperfusion injury occurring in infarction [47, 77–79]. It has the capability to inhibit caspase-3 activity, reducing H/R induced apoptosis and enhancing cell resistance to oxidative stress [47]. It suppressed TLR4 signaling (by blocking the pathway including Toll-like receptors) and lowered inflammatory cytokine secretion, which inhibited development of inflammation within the damaged cardiac muscle [77]. The protective effect of HSYA consisted in its inclusion in the course of the PI3K/Akt/hexokinase II signaling pathway to activate hexokinase II proteins, which consequently reduced cell apoptosis [79]. Intravenous administration of carthamine yellow (CY) also has favorable impact on the treatment of the effects of acute myocardial infarction. It supported angiogenesis, which increased the number of unobstructed capillaries in the area adjacent to the infarction site and favored revascularization and restoration of normal function of the cardiac muscle [80]. Moreover, research confirmed positive effect of hydroxysafflor C (HSYC) on the prevention of cardiovascular diseases [52].

Substances isolated from safflower flowers have therapeutic effect on blood pressure problems. HSYA reduced systolic pressure in the left ventricle in rats with pulmonary hypertension [78]. This pigment produced dilation of vessels in the mesenteric artery in rats which resulted in reduced blood pressure thanks to elevated transport of Ca^2+^ ions to endothelial cells, eNOS phosphorylation, and nitric oxide synthesis [81]. The strongly dilating effect of HSYA on vessels is further related to its activation of the KV channel in the pulmonary vascular smooth muscle cells [82]. Additionally, positive impact of carthamine yellow (CY) on the blood coagulation process was revealed [62, 83]. CY reduced blood viscosity, plasma viscosity, and erythrocyte aggregation index, which resulted in reduction in blood pressure. Decrease in hematocrit and platelet aggregation was observed with increased CY dose. This may pose a chance to prevent embolisms by increasing blood liquidity. The study authors pointed to the fact that using CY as a pigment in foods may pose risk in the case of persons with hemorrhages, but they estimated it to be minor [83].

### 4.11. Digestive System

HSYA inhibited liver cell fibrosis [84, 85] resulting from induction of apoptosis of stellate cell responsible for the development of disease by blocking activation of expression of the genes regulated by ERK1/2 (including Bcl-2, cytochrome C, caspase-9, and caspase-3 [84]) and thanks to PPAR activation, increasing activity of antioxidative enzymes, increasing expression of PPAR and MMP-2, reducing expression of TGF-1 and TIMP-1, and lowering *α*-SMA level [85]. In the organisms of aging mice and mice exposed to pathologic changes, HSYA fulfilled protective role for the liver and other organs, reducing the level of mRNA and the amount of cyclin-dependent protein kinase inhibitor p16 [86]. Similarly, the extract obtained from safflower leaves can fulfill protective role towards liver exposed to injury due to administration of antituberculous drugs (this pigment produced substantial reduction of AST, ALT, and ALP parameters and total bilirubin). Studies on methanol extract further revealed presence of lupeol (a triterpenoid of anti-inflammatory and antineoplastic significance) and *β*-sitosterol (phytosterol producing, among others, poorer absorption of cholesterol in the digestive tract [87]). A herbal blend including dried safflower flowers and *Salvia miltiorrhiza* root administered as an injection (Danhong injection) alleviated gastric mucosal lesions caused by administration of salicylic acid, even with prolonged exposure to the drug. It reduced pepsin production and reduced the level of reactive oxygen species in the gastric mucosa [88].

### 4.12. Neoplastic Diseases

HSYA inhibits the development of cancers [45, 51, 89–93]. It inhibited angiogenesis of liver cancer by blocking the ERK/MAPK [89, 94] and NF/_K_B HSYA signaling pathway in mice [89] and via inhibiting p38MAPK phosphorylation [91]. Administration of HSYA enhanced spleen and thymus indicators and immune system function [89] and resulted in reduced viability, proliferation, and migration of HepG2 tumor cells [91]. By affecting the NF-*κ*B signaling pathway, HSYA also inhibited the development of malignant esophageal cancer cells: their proliferation and migration were blocked and accelerated apoptosis was observed (blockage of ICAM1, MMP9, TNF-*α*, and VCAM1 expression and stimulation of phosphor-nuclear transcription factor kappa B p65 expression) [92]. Similarly, positive effect was observed following application of a herbal mixture containing, among others, *Carthamus tinctorius* flowers on the level of HFP, ALT, and TBIL hepatic indicators. This multicomponent product inhibited development of hepatocellular carcinoma (HCC) in humans and increased overall survival, as well as minimized the risk of complications [93].

HSYA reversed drug resistance to chemotherapy drugs of ovarian carcinoma cells in mice [45, 90] by unblocking MAPK signaling pathways in resistant cells [45]. Thanks to the expression of WSB1 gene, the tumor cell proliferation was also inhibited [90]. Furthermore, the pigment exhibited anti-inflammatory and antioxidative action, affected survival rate of cells, and inhibited tumor angiogenesis [45].

HSYB, an isomer of HSYA, inhibited proliferation of breast cancer cells (MCF-7) in humans and reduced survival rate and proliferation of tumor cells by blocking their cellular cycle in S phase. Increased apoptosis of tumor cells was linked to reduced level of cyclin D1, cyclin E, CDK2, p-PI3K, PI3K, AKT, and p-AKA proteins in MCF-7 cells and reduced level of Bcl-2 [51].

### 4.13. Adverse Symptoms

Doses up to 2000 mg/kg body weight of carthamus red are safe (no toxicity was found [95]); however, with long-term oral administration of higher concentrations of safflower flower extracts (200 mg/kg for 35 days), changes in the functioning of the male reproductive system [96, 97], pharyngitis, and nosebleeds [98] were observed. Injection of safflower extract induced allergy reactions [99, 100], and with intraperitoneal administration of HSYA, slight changes in the functioning of the kidneys (at a dose of 180 mg/kg [101]) and liver [102] were noted.

## 5. Applicability of Safflower Bioactive Substances in Food Industry

Importance of *C. tinctorius* is primarily linked to the commercial use of seed and flower petals. Seeds are mainly used for the production of edible oil and for feed purposes, whereas flower petals are used to obtain dyes applied in apparel, food and cosmetic industry, medicine, and in painting ([32, 103], Henry and Francis 1996 by [104], [105, 106]), while above-ground parts of plants are used to produce animal feed [107].

**Figure 2:**
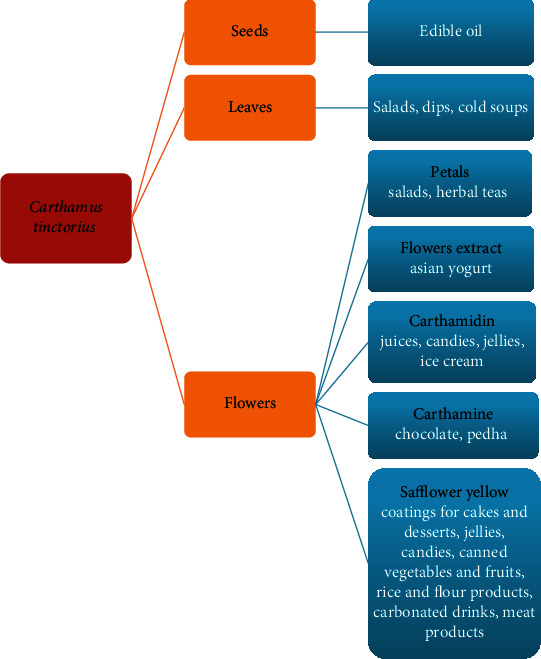
The use of safflower flowers and its components in food production.

Due to the high content of carotene, riboflavin, and vitamin C in the green parts, in India this plant is cultivated as leaf vegetable [108]. Safflower leaves are characterized by perceptible bitter taste [54]. In order to retain the valuable leaf components, they should not be subject to prolonged treatment in high temperature, suggesting that they can (after a possible brief blanching) constitute an interesting addition to fresh salads, dips, and cold soups, adding a bitter flavor (Figure 2).

Safflower flowers also carry a potentially high significance for food production, because they can constitute an ingredient enriching meals with nutrients. Flower petals contain all necessary amino acids, except tryptophan. Flowers of thornless cultivars are popular already: they have been shown to be rich in protein, sugars, calcium, iron, magnesium, and potassium. By using these properties, teas, whose main ingredient are *C. tinctorius* petals, were composed and popularized in China and India (Singh (2005a) by [10]). However, these petals can comprise an interesting ingredient of fresh salads, which does not only enrich the sensory values of products, including flavors (associated with volatile oils present in the flowers) or values linked to the bright coloration of the petals. Such petals could also constitute an additional source of valuable bioactive dietary nutrients (Figure 2). Safflower petals were used on a mass scale to obtain pigments for food products, but when less expensive synthetic pigments were made popular, the use of natural colorants was markedly lowered. This approach changed with the introduction of legal regulations on the substances authorized for use in food production in many countries, when the interest in natural sources of colorants increased again [10].

Thus far, mainly carthamine and carthamidin have been used in food production (Figure 2). Carthamine, due to its poor water solubility, was used to dye chocolate, while carthamidin can be found in colorful juices, jellies, and candies (after [103]). Carthamine is unstable in aqueous solutions and decomposes very easily at elevated temperature and in alkaline solutions (Fatahi et al. (2009) by [109]); therefore, a method for stabilizing this pigment was developed using natural ionic liquids and deep eutectic solvents, which are natural primary metabolites [109]. These are usually sugars and sugar alcohols, amino acids and amines, and organic acids, which possess several hydroxyl, carboxyl, or amine groups [110]. Such solutions protect carthamine against the negative impact of light, temperature, and prolonged storage [109]. The use of natural dyes obtained from safflower flowers (yellow safflower extract) in food production is allowed in the EU and Asia, but banned in the US [111], although producers of safflower yellow recommend them for use in meat preparations, cake coatings and desserts, jellies, candies, and canned vegetables and fruit as well as flour and rice products and carbonated drinks [112].

The applicability of carthamidin as an ice cream dye was investigated. Addition of this pigment had positive effect on sensory evaluation and chemical composition of products. The highest sensory acceptability is characterized by ice cream containing 0.06 ml of carthamidin; increasing and decreasing the content of this substance deteriorated results of sensory evaluation, particularly for flavor, color, and texture of product. Addition of the pigment caused a slight increase in the moisture and the content of protein, lipids, carbohydrates, and ash. The authors of this study pointed to the health benefits of using this natural pigment [30]. The production technology of Pedha was also developed and tested with the addition of carthamine as the pigment that gives the yellow color of this traditional Indian sweet snack [113]. The addition of carthamine resulted in a minor increase of moisture and protein and ash content and minor decrease in fat and lactose content, as well as acidity. The percentage sucrose content when 5% addition carthamine was used was slightly lower than in the product without the pigment, but higher when the dose constituted 10% of product's mass. The highest score of sensory assessment was given to the product containing 10% carthamine (evaluated color, taste, flavor, and texture). Concentrate obtained from safflower was also used to produce Asian-style yogurt, a product with the addition of lychees and elderberry extract (Figure 2) [114].

However, the dyes contained in the safflower flowers have not been used in food production to take advantage of their health-promoting nature. The exception is herbal teas containing whole dried flowers. Introduction of HSYA to food production would be highly beneficial from the consumers' standpoint. This flavonoid is widely applied in medicine, and its addition to food products at the production stage, naturally at doses lower than therapeutic, would greatly improve their health-promoting value. Such products would be of substantial significance for the reduction of oxidative stress within different tissues and organs, and they could exhibit prophylactic action towards cardiovascular diseases and neoplastic diseases, among others.

An additional aspect favoring the addition of HSYA to foods is its beneficial impact on the reduction of adipose tissue and body weight following oral administration associated with its effect on the composition of intestinal microorganisms and cellular metabolism [67], on the inhibition of formation of new adipose cells [68] and enhancement of liver function [50]. Physical properties of HSYA (density 1.9 ± 0.1 g/cm^3^, boiling point at 1015.8 ± 65°C at 760 mmHg, and ignition point at 334.0 ± 27.8°C; after [115]) indicate that it is not difficult for technological application. The only problem for HSYA application in food production is its poor absorption from the gastrointestinal tract: the absolute absorption was only 1.2% [116]. However, studies have shown that in order to improve absorption of HSYA, it should be used in the form of a water-in-oil microemulsion: then, the availability of this pigment for the organism increases by 1937%, and digestion of the microemulsion occurs thanks to pancreatic lipase (in this form up to 60% of the microemulsion is digested within 1 hour) [117]. This observation opens the possibilities of enriching food products with the suitable consistency with HSYA: i.e., spreads and vegetable or fruit pastes. The process of microencapsulation of ingredients desired in food is also a great opportunity for HSYA.

Furthermore, research conducted on rats provided evidence that after intravenous administration of HSYA the presence of metabolites of the pigment was found in blood plasma, bile, urine and faeces, but these were not toxic values [116]. It is also important to note that the pigment did not accumulate in the organism: it was excreted mainly in urine [118] and faeces, and the half-life of its metabolites in blood was only 6 hours. After this time, 90% of HSYA dosage was eliminated from the organism [116].

## 6. Conclusions

The wide commercial use of safflower means that the demand for its flowers is steadily increasing. In China, 1800–2600 MT of flowers were produced annually at the beginning of the 21st century [119]. Due to the possibility of using almost all parts of the plant, the profit from the cultivation of this plant is continuously growing (Sawant et al. (2000) by [10]). The health-promoting applicability of safflower increases with the progressing knowledge on its chemical composition.

## Figures and Tables

**Table 1:** The main components of the safflower flower extract.

Compound	Structure	Molecular formula	Molecular weight (g/mol)	Color (and form)	Source of information
Carthamine (syn. cartamin)	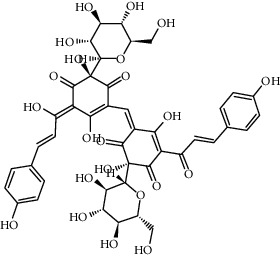	C_43_H_42_O_22_	910.8	Red powder	[120]
Precarthamine	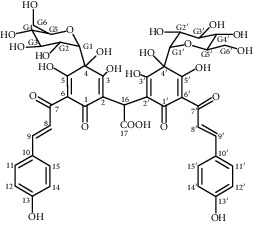	C_44_H_44_O_24_	956.83	Yellow	[32]
Carthamidin	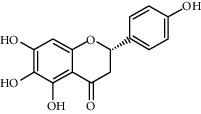	C_15_H_12_O_6_	288.25	Yellow	[120]
Carthamusin A	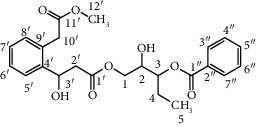	C_24_H_28_O_8_	444.474	White crystals	[17]
Cartormin	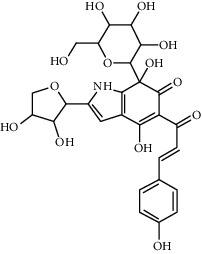	C_27_H_29_NO_13_	575.5	Yellow crystals	[120]
Isocartormin	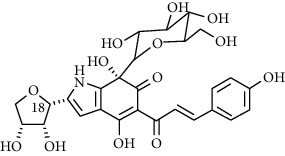	C_27_H_29_NO_13_	575.5	Yellow needle crystals	[29]
Hydroxysafflor yellow A (HSYA)	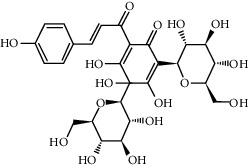	C_27_H_32_O_16_	612.5	Yellow	[120]
Anhydrosafflor yellow B (AHSYB)	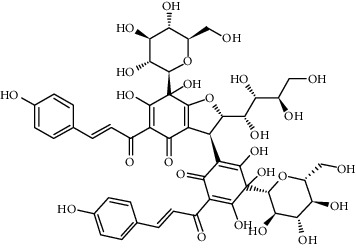	C_48_H_52_O_26_	1044.9	Yellow	[120]
Safflomin C	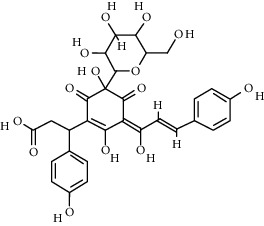	C_30_H_30_O_14_	614.5	Yellow powder	[121]
Isosafflomin C	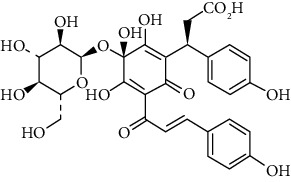	C_30_H_30_O_14_	614.5	Yellow	[122]
Safflor yellow A	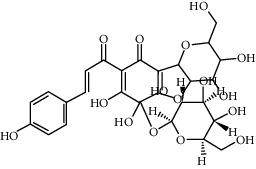	C_27_H_30_O_16_	610.5	Yellow	[120]
Safflor yellow B (syn. safflomin B)	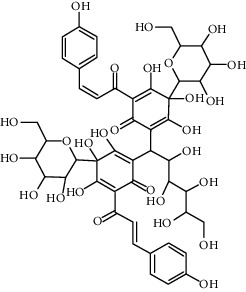	C_48_H_54_O_27_	1062.9	Yellow	[120]
Saffloroside	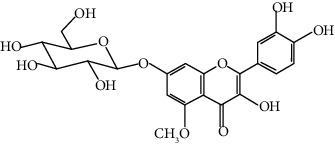	C_22_H_22_O_12_	478.403	Yellow crystals	[37]
Azaleatin	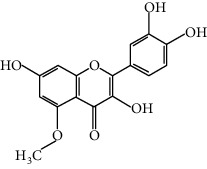	C_16_H_12_O_7_	316.26	Yellow crystals	[120]
Cinaroside	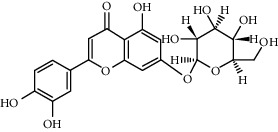	С_21_Н_20_О_11_	448.377	Light-yellow crystals	[120]
Guanosine	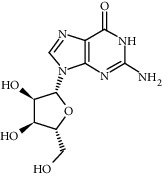	C_10_H_13_N_5_O_5_	283.24	White crystalline powder	[120]
Kaempferol	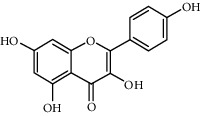	C_15_H_10_O_6_	286.24	Light yellow powder	[120]
Kaempferol 3-*O*-*β*-rutinoside (syn. nicotiflorin)	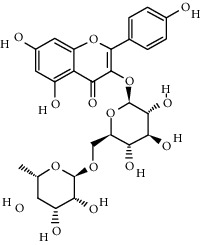	C_27_H_30_O_15_	594.5	Yellow, powder or crystals	[121]
Luteolin	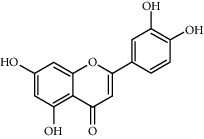	С_15_Н_10_О_6_	286.2	Yellow crystals	[120]
Luteolin 5-methyl ether (syn. 5-O-methylluteolin)	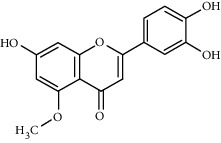	C_16_H_12_O_6_	300.3	Yellow powder	[120]
Quercetin	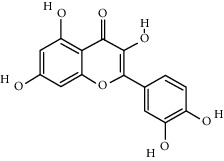	C_15_H_10_O_7_	302.2	Yellow needle crystals	[121]
Stigmasterol	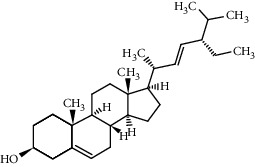	C_29_H_48_O	412.7	White powder	[120]
Syringin	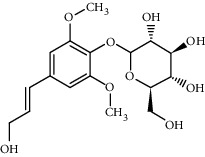	C_17_H_24_O_9_	372.4	White crystals	[120]
Tinctormine	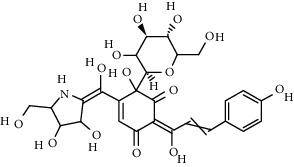	C_27_H_31_NO_14_	593.5	Yellow powder	[121]
*β*-Daucosterol (syn. sitogluside)	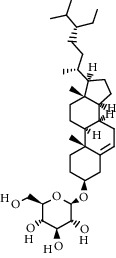	C_35_H_60_O_6_	576.8	White powder	[121]
6-Hydroxykaempferol 3-O-*β*-D-rutinoside	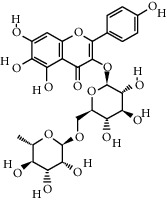	C_27_H_30_O_16_	610.5	Yellow powder	[121]
6-Hydroxykaempferol-3-*O*-*β*-D-glucoside-7-*O*-*β*-D-glucuronide	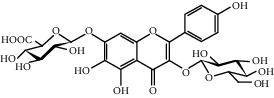	C_27_H_28_O_17_	624.5	Yellow	[38]

**Table 2:** Selected studies of antioxidant activity of substances present in safflower flowers.

Substance	Research object	Type of test	Parameter determined	Tested concentrations	Results	Authors
HSYA^∗^	*In vitro*: tyrosinase, l-DOPA	Tyrosinase activity assay	Change in absorbance per min at 475 nm	0, 0.5, 1.5, and 2.5 mM HSYA	Inhibition of l-DOPA oxidation, reduction of tyrosinase activity	[43]

HSYA	*In vitro*: INS-1 (rat insulinoma cells)	CAT kit	CAT mRNA level	800 mM HSYA	Increase of SOD, GSH-Px, and CAT levels; decrease of ROS and MDA levels	[44]
		GSH-Px kit	GSH-Px mRNA level			
		SOD kit	SOD mRNA level			
		MDA kit	MDA level			

HSYA	*In vivo*: mice	Lipid peroxidation MDA assay kit	MDA level	3.5 mg kg^−1^ HSYA in 0.1 ml; injection	Control: approx. 21 nmol/mg; DHEA+ HSYA = approx. 22 nmol/mg	[46]
		GSH and GSSG assay kit	GSH/GSSG ratio		Control: approx. 7.7; DHEA+ HSYA = approx. 6.4	
		SOD assay kit	SOD activities		Control: approx. 246 U/mg; DHEA+ HSYA = approx. 200 U/mg	
		GSH-Px assay kit	Activities of GSH-Px		Control: approx. 600 mU/mg; DHEA+ HSYA = approx. 500 mU/mg	
		CAT assay kit	Activities of CAT		Control: approx. 59 U/mg; DHEA+ HSYA = approx. 38 U/mg	

HSYA	*In vivo*: rats	Commercial assay kits, fluorescence spectrophotometry	*SOD* activity	5 mg/kg HSYA; intraperitoneally injected	Control = 199; HSYA = 125	[47]
			Content of MDA		Control = 7; HSYA = 12	

HSYA, carthamine	*In vitro*: HepG2 cells and 3T3-L1 adipocytes	MDA assay kit	MDA level	200 mg/kg/d SY or HSYA; injection	Increase of SOD activity and MDA level (in a high-fat diet group)	[50]
		SOD assay kit	SOD activities			

HSYC	*In vitro*: H9c2 (rat pheochromocytoma cells)	Antioxidative effects against H_2_O_2_-induced cytotoxicity	Cell viability	60 *μ*g/ml HYSC	71% of control; control: vitamin C (1.1 mg/ml)	[52]

Methanol extract of safflower flowers	*In vivo*: rats	FRAP	Optical density at 700 nm	20 mg/ml, 40 mg/ml, and 80 mg/ml	0.749; 1.155; 1.532 (respectively)	[49]
		DPPH	Absorbance at 517 nm; IC_50_	0.1 ml solution of different concentrations of extract	IC_50_ = 0.36	

Safflower flower extract, HSYA, SYA	*In vitro*: HuDe (human dermal fibroblasts)	ORAC	Fluorescence emission intensity at 530 nm	Water extract (5%), HYSA, SYA	Total antioxidant activity 130.2 ± 12.3 mmol TE/100 g; Trolox index HSYA = 7.1 ± 0.3; Trolox index SYA = 2.1 ± 0.1	[48]
		DPPH	Absorbance at 517 nm; IC_50_		IC_50_extract = 13.4 ± 1.0 (*μ*g GAE/ml); IC_50_HSYA = 7.3 ± 1.2 (*μ*g HSYA/ml); IC_50_SYA = 30.3 ± 2.9 (*μ*g SYA/ml)	

^∗^Symbols: CAT: catalase; DHEA: dehydroepiandrosterone; GSH/GSSG ratio: reduced glutathione/oxidized glutathione; GSH-Px: glutathione peroxidase; HSYA: hydroxysafflor A; HYSB: hydroxysafflor B; HYSC: hydroxysafflor C; MDA: malondialdehyde; SOD: superoxide dismutase; SYA: safflor A.

**Table 3:** Studies on the use of HSYA for medical purposes (from 2018 to 2020).

Test organism	Type of test/parameter	Concentration/dose	Authors
*In vitro*: LAD2 (human mast cells) and MPMCs (mouse peritoneal mast cells)	Intracellular Ca^2+^ mobilization assay/imaging with excitation at 488 nm	50 *μ*M, 100 *μ*M, and 200 *μ*M HSYA (pH 7.4)	[57]
*In vitro*: LAD2 (human mast cells)	MTT assay (cell viability assay)/absorbance at 490 nm	1 *μ*M, 10 *μ*M, 50 *μ*M, 100 *μ*M, 200 *μ*M, and 400 *μ*M	
	*β*-Hexosaminidase release assay/absorbance at 405 nm	50 *μ*M, 100 *μ*M, and 200 *μ*M	
	ELISA kits (human chemokine array kits)/measurements of cytokines (levels of TNF-*α*, interleukin- (IL-) 8, and MCP-1)		
	LC-ESI-MS/MS (liquid chromatography-electrospray ionization-tandem mass spectrometry)/histamine release assay		
*In vitro*: protein extracted from LAD2 cells	Western blot analysis (ECL kit)/protein expression (transillumination)		
*In vivo*: mice	Hindpaw swelling and extravasation assay/optical density at 620 nm	0, 2.5 mg/kg, 5 mg/kg, 10 mg/kg HSYA in saline	

*In vivo*: mice	ELISA kits/assay the levels of testosterone, estradiol, progesterone, luteinizing hormone, follicle-stimulating hormone, anti-Müllerian hormone (AMH)	3.5 mg kg^−1^ HSYA in 0.1 ml, injection	[46]

*In vitro*: INS-1 (rat insulinoma cells)	Cell viability assay/absorbance at 570 nm	200 *μ*M, 400 *μ*M, 800 *μ*M, and 1600 *μ*M	[44]
	Western blot analysis/protein expression (transillumination, band densities)	800 *μ*M	
	Insulin ELISA kit/glucose stimulated insulin secretion		

*In vivo*: rats	Anthrone method/hepatic glycogen in the liver	120 mg/kg, for 8 weeks	[59]
	WST-8 method/glycogen synthase		
	Fasting blood glucose/glucometer test		
	Oral glucose tolerance test/glucometer test (0, 30, 60, and 120 minutes after oral administration with glucose solution)		
	Assay kits/fasting blood insulin, triglycerides, total serum cholesterol, high-density lipoprotein cholesterol, low-density lipoprotein cholesterol in serum		
	Western blot analysis/protein expression		

*In vitro*: HK-2 (renal tubular epithelial cells)	Western blot analysis/protein expression	10 mg/ml HSYA	[60]
	Cellular immunofluorescence assay/photographing (fluorescent inverted microscope)		
*In vivo*: rats	ELISA kit/interleukin-6 levels, tumor necrosis factor-*α* level	Doses of 10 mg/kg daily, intragastrically, for 6 weeks	
	RT-qPCR assay (PCR)/relative expression level of miRNA-140-5p		
	BCA method (based on T-AOC and MDA kit)/protein concentrations		

*In vitro*: PC12 (pheochromocytoma cell line)	MTT assay/cell viability	5 *μ*M, 10 *μ*M, 20 *μ*M, 40 *μ*M, and 80 *μ*M	[63]
	Total mRNA (amount and purity)/absorbance at 260/280 nm	10 *μ*M	
	Western blot/protein expression		
*In vitro*: b.End3 (mouse brain microvascular endothelial cells)	Immunofluorescence assay/immunofluorescence (confocal microscopy)	20 *μ*M	
*In vivo*: rats	Therapeutic effect in vivo/infarct area, ratio of infarct volume to whole brain tissue	10 mg/kg, 20 mg/kg	

*In vitro*: NSCs (neural stem cells of rats)	CCK-8 assay/absorbance at 450 nm	1 *μ*M, 5 *μ*M, and 10 *μ*M	[65]
	EdU assay/cell proliferation rate		
	Cell apoptosis/BD FACSCalibur flow cytometry		
	Western blot analysis/protein expression		

*In vivo*: mice	Hanging wire test/hanging time	20 mg/kg/d, for 28 days	[66]
	Western blot analysis/protein expression		

*In vivo*: mice	IPGTT (intraperitoneal glucose tolerance test), IPITT (intraperitoneal insulin tolerance test)/glucose and insulin tolerance tests	200 mg/kg/d HSYA, for 10 weeks	[50]
	2−DDCt method/expression of antioxidant enzymes in the liver and adipose tissue		
*In vitro*: HepG2 cells and 3T3-L1 adipocytes	2−DDCt method/expression of antioxidant enzymes	3T3-L1 adipocytes: 10, 50, and 100 mg/l HSYA for 24 h; HepG2 cells: 10, 50, and 100 mg/l SY^∗^ for 24 h	
	ALP activity assay/percentage of ALP activity		
	Caspase colorimetric assay kit/caspase-3 activity assay		
	Annexin V-FITC/IP staining kit/percentage of apoptotic cells		
	Western blot analysis/protein expression		
	HOP^∗^ and HOM^∗^ concentration in serum		

*In vitro*: HSAEC1-KT HSAECs (human small airway epithelial cells)	ELISA kits/concentrations of inflammatory cytokines (IL-6, IL-1*β*, and TNF-*α*)	9, 27, and 81 mmol/l	[74]
	Western blot analysis/protein expression		
	Calcium-sensitive fluorescent probe Fluo-3/AM/intracellular calcium ion concentration		
	Dual-luciferase reporter assay/transcriptional activities of NF-*κ*B and AP1		

*In vivo*: rats	Double-staining method (with Tcc and Evans blue stains)/infarct size	5 mg/kg HSYA; intraperitoneally injected	[47]
	ELISA kits/cTnI, IL-6, and LdH levels		
	Flow cytometry analysis, TUNEL (terminal deoxynucleotidyl transferase-mediated dUTP nick-end labeling)/apoptosis level		
*In vitro:* H9c2 (rat cardiomyoblast cells)	ELISA/caspase-3 activity	20 *μ*M	
	CCK-8/cell viability		
	Western blot analysis/protein expression		
	Span diagnostic reagent kit/serum glutamic oxaloacetic transaminase		
	Span diagnostic reagent kit/serum alkaline phosphatase		
	Agappe diagnostic kit/serum total bilirubin		

*In vivo*: mice	SP kit (MMP-2, MMP-9, and COX-2)/immunohistochemical analysis	1.125 mg/kg, 2.25 mg/kg, intraperitoneally, for 14 days	[91]
*In vitro*: H22 (murine hepatoma cells), HepG2 (human hepatocellular carcinoma cells)	Growth inhibition assay—CCK-8/rate of growth inhibition (IC_50_ 80 *μ*M for 72 h)	40, 60, 80, 120, and 160 *μ*M, for 24, 48, and 72 h	
	Western blot analysis/protein expression		
	Clonogenic assay/optical density at 570 nm		
	Wound healing assay/cell migration, via Image-Pro Plus		

*In vitro*: KYSE-30 cells (esophageal cancer cells)	CCK-8 assay/proliferative activity (optical density of cells)	0,1, 1, 10, 20, and 50 *μ*M, for 24, 48, and 72 h	[92]
	Microscopic measurement of the number of cells/cell invasion and cell migration assays	20 *μ*M, for 24 h	
	Flow cytometric analysis of cell apoptosis/apoptotic rate of cells		
	Western blot analysis/protein expression		
	Serum levels of AFP, ALT, TBIL, and ALB/automatic analyzer		

*In vitro*: A2780/DDP (ovarian carcinoma cells)	Cytotoxicity assay (CCK-8)/cell survival rates	1 mg/ml	[45]
	RTCA test (real-time cellular analysis)/monitoring cell proliferation		
	Flow cytometry (Guava EasyCyte Plus)/evaluation of apoptosis	1 mg/ml, for 24	
*In vivo*: mice	Electronic digital caliper/weekly measurement of tumor size and volume	1.1 g/kg body weight; once every three days, for five weeks	
	Western blot analysis/protein expression		

*In vitro*: SKOV-3 (ovarian carcinoma cells)	Cell proliferation assay kit/cell proliferation	10, 20, 50, 100, and 150 mg/l, for 72 h	[90]
	CellTiter-Blue Cell Viability kit/cell viability assay	10, 20, 50, 100, and 150 mg/l, for 48 h	
	Western blot analysis (protein concentration assay kit)/protein expression	10, 20, 50, 100, and 150 mg/l	

n.d.: no date; SY: carthamine; HOP: hydroxyproline; HOM: hexosamine; CCK-8: cell counting kit.

**Table 4:** Studies on the use of the substances contained in safflower flowers, except HSYA, for medical purposes (from 2018 to 2020).

Substance or plant	Test organism	Type of test/parameter	Concentration/dose	Authors
Safflower yellow (SY)	*In vitro*: HUVEC-12 (human umbilical vein endothelial cells), BMSCs (bone marrow stromal cells)	Transwell and tube formation assay/migration and angiogenesis of HUVEC-12 cells	4.5, 9.0, and 18 *μ*g/ml	[69]
		Western blot analysis/protein expression		

Polysaccharides isolated from safflower	*In vitro*: calvarial osteoblast cells of rats	MTT assay/cell proliferation (percentage of cell viability)	25, 50, and 100 *μ*g/ml, for 24, 48, and 72 h	[70]
		ALP activity assay/percentage of ALP activity		
		Caspase colorimetric assay kit/caspase-3 activity assay		
		Annexin V-FITC/IP staining kit/percentage of apoptotic cells		
		Western blot analysis/protein expression		

(1→3)-linked *β*-d-glucan	*In vivo*: rabbits	Western blot analysis/protein expression	25, 100, and 200 mg/kg, for 60 days	[72]
		HOP (hydroxyproline) and HOM (hexosamine) concentration in serum		

Methanol extract of flowers	*In vivo*: rats	Histological studies/examination of pancreatic tissue	Doses 200 mg/kg daily, intraperitoneally, for 4 weeks	[49]

Methanol extract of *C. tinctorius* leaves	*In vivo*: rats	Span diagnostic reagent kit/serum glutamic pyruvic transaminase level	100, 200, and 400 mg/kg body, for 24 days; oral route	[87]
		Span diagnostic reagent kit/serum glutamic oxaloacetic transaminase		
		Span diagnostic reagent kit/serum alkaline phosphatase		
		Agappe diagnostic kit/serum total bilirubin		

LDF (Chinese herbal formula)	*In vivo*: patients with advanced HCC (liver cancer)	Overall survival and time to progression	100 ml/time, three times a day	[93]
		Serum levels of AFP, ALT, TBIL, and ALB/automatic analyzer		

*Carthamus oxyacantha*	*In vitro*: *Escherichia coli*, *Pseudomonas aeruginosa*, *Salmonella typhi*, and *Staphylococcus aureus* strains	Minimum inhibitory concentration (MIC)/the minimum inhibitory concentration	Extract: 1.0 mg/ml, 2.0 mg/ml, 3.0 mg/ml, 4.0 mg/ml, and 5.0 mg/ml	[55]
		Well diffusion method/zone of inhibition against pathogens growth	200 *μ*l of plant extract	
	*In vivo*: mice	Castor oil-induced diarrhea; magnesium sulfate-induced diarrhea/% inhibition of defecation, latency	Methanol extract: 200 mg/kg, 400 mg/kg	
		WST-8 method/glycogen synthase		
		Fasting blood glucose/glucometer test		
		Oral glucose tolerance test/glucometer test (0, 30, 60, and 120 minutes after oral administration with glucose solution)		
		Assay kits/fasting blood insulin, triglycerides, total serum cholesterol, high-density lipoprotein cholesterol, low-density lipoprotein cholesterol in serum		
		Western blot analysis/protein expression		

**Table 5:** Action mechanisms by safflower substances.

Activity	Result of mechanism	Mechanism	Authors
Prevention of anaphylaxis	(i) Inhibition of mast cell degranulation (ii) Reduction the activation of the PLC*γ*-PKC-IP3 signaling pathway	(i) Inhibition of Ca^2+^ flow (ii) Inhibition of MCP-1, IL-8, *β*-hexosaminidase, HA, and TNF-*α* release (iii) Inhibition of phosphorylation of PLC*γ*1, IP3R, PKC, Akt, P38, and Erk1/2	[57]

Alleviation of polycystic ovary syndrome	(i) Reduction of cysts (ii) Regulation of the hormonal balance (iii) Restoration of the ovulation cycle	(i) Reversion of the expression of genes Star, Hsd3b1, Cyp11a1 (increase), and Cyp19a1 (reduction) (ii) Increase in antioxidant enzyme activities (SOD, GSH-Px, and CAT) (iii) Regulation of the level of T, E2, FSH, P4, and AMH and the ratio of LH/FSH in serum (iv) Reduction of MDA level and enhanced GSH content and GSH/GSSG ratio	[46]

Antitumor effects	(i) Induction of cisplatin sensitivity by JNK and P38 MAPK signaling pathway	(i) Increase in P-JNK and P-38 levels	[45]
	(i) Inhibition of cancer cell proliferation	(i) Inhibition of Skov3 cell proliferation (ii) Reduction of WSB1 expression (iii) Inhibition of Erk1/2 expression and Erk phosphorylation	[90]
	(i) Inhibition of cancer cell proliferation (ii) Induction of cancer cell apoptosis	(i) Inhibition of the MCF-7 cell cycle at the S phase (ii) Reduction of CDK2, cyclin D1, and cyclin E levels (iii) Reduction of p-PI3K, PI3K, AKT, and p-AKT levels	[51]
	(i) Inhibition of tumor angiogenesis	(i) Inhibition of p38 MAPK phosphorylation (ii) Reduction of MMP-2 and MMP-9 levels (iii) Reduction of COX-2 expression by p38MAPK/ATF-2 signaling pathway (by inhibition of p38MAPK phosphorylation) (iv) Increase of the caspase-3 cleavage in tumor cells	[91]
	(i) Induction of autophagy in cancer cells by regulating Beclin 1 and ERK expression	(i) Increase in Beclin 1 and LC3-II expression in tumor cells (ii) Reduction of phosphorylated ERK1/2 expression and p62 level in tumor cells	[94]
	(i) Induction of apoptosis of tumor cells by regulating the NF-*κ*B signaling pathway (inhibition of the tumor growth)	(i) Inhibition of the expression of ICAM1, MMP9, TNF-*α*, and VCAM1 (ii) Increase in the expression of p-I*κ*B*α* and pP65	[92]

Alleviation of damage and brain injuries	(i) Inhibition of the activation of the pyroptotic pathway and apoptosis of injured nerves (ii) Activation of damage mitigating factor	(i) Reduction of cytokine expression (NLRP3, ASC, caspase-1, GSDMD, IL-1*β*, IL-18, LDH, NF-*κ*B, and p-p56) (ii) Changes in activation of the NF-*κ*B signaling pathway	[63]
	(i) Reduction of the apoptosis and autophagy of neural stem cells by modulation of the p38/MAPK/MK2/Hsp27-78 signaling pathway (ii) Stimulation of the cell proliferation	(i) Reduction of p38 and Hsp27-78 phosphorylation and MK-2, Bax, cleaved caspase-3, LC3-II, and mTOR phosphorylation expression (ii) Increase in Bcl-2 and p62 expression	[65]
	(i) Inhibition of dopamine synthesis (ii) Promotion *α*-syn clearance by regulating autophagy	(i) Increase in the formation of autophagosomes (ii) Increase of TH, p-JNK1/JNK1, Beclin 1, Atg7, Atg12-5, and p-Bcl-2/Bcl-2 expression and the LC3-II/LC3-I ratio (iii) Reduction of *α*-syn expression	[66]

Alleviation of diabetes complications	(i) Inhibition of JNK/c-Jun signaling pathway (ii) Alleviation of oxidative damage	(i) Inhibition of p-JNK and p-c-Jun activation (ii) Reduction of phosphorylation of JNK and c-Jun (iii) Reduction of cleaved parp and cleaved caspase-3 levels	[44]
	(i) Promotion of PI3K and Akt activation (ii) Inhibition of the apoptosis of pancreatic *β*-cells	(i) Increase of PI3K, AKT, and p-AKT expressions (ii) Increase in contents of hepatic glycogen and glycogen synthase in the liver	[59]
	(i) Reduction of renal fibrosis (ii) Regulation of the TLR4/NF-*κ*B(p65) pathway and miRNA-140-5p level	(i) Increase of miRNA-140-5p mRNA, BG, 24 h UP, TC, TG, T-AOC, MDA, IL-6, TNF-*α*, TLR4, NF-*κ*B(p65), NLRP3, Notch2, and Col-IV	[60]

Protection of the digestive system	(i) Protection of the liver and other organs against aging	(i) Increase of CAT, GSH-Px, MDA, and SOD activities (ii) Reduction of the mRNA, protein level of cyclin-dependent kinase inhibitor p16 and phosphorylation of pRb (iii) Increase in CDK4/6 protein expression	[86]
	(i) Protection of the liver against damage	(i) Reduction of ALT, ALP, AST, and total bilirubin levels	[87]

Protection and treatment of cardiovascular diseases	(i) Change in platelet activation pathway	(i) Regulation of core genes: PRKACA, PIK3R1, MAPK1, PPP1CC, PIK3CA, and SYK	[76]
	(i) Inhibition of activation of the JAK2/STAT1 pathway	(i) Inhibition of caspase-3 activity (reduction of H/R-induced apoptosis) (ii) Reduction of Janus kinase 2 (JAK2)/signal transducer and activator of transcription 1 (STAT1) activity (iii) Reduction of releases of cTnI, IL-6, and LdH (iv) Change of expression levels of Bcl-2-associated X protein, Bcl-2, cleaved caspase-3, Fas ligand, and tumor necrosis factor receptor superfamily member 6 (Fas)	[47]
	(i) Effect on vasodilation	(i) Inhibition of the PKA and NO production (ii) Activation of p-eNOS expression (iii) Change of the influx of Ca^2+^ (TRPV4-dependent)	[81]

Protection of skeletal system	(i) Regulation of pVHL/HIF-1*α*/VEGF pathway (ii) Increase in angiogenesis and bone cell differentiation (iii) Inhibition of HIF-1*α* expression	(i) Increase of ALP, Ang-2 (Angiopoietin-2), HIF-1*α* (hypoxia inducible factor-1*α*), OPN-1 (osteopontin-1), Runx2 (runt-related transcription factor 2), and VEGF (vascular endothelial growth factor) levels (ii) Inhibition of SY-induced proliferation, migration, and angiogenesis	[69]
	(i) Increase in osteoblast differentiation (ii) Inhibition of osteoblast apoptosis	(i) Inhibition of caspase-3 activity (change in caspase-3-dependent signaling pathway)	[70]

Protection of the respiratory system	(i) Inhibition of the platelet activating factor in the airway epithelium (ii) Reduction of inflammation	(i) Changes in the expression of interleukin- (IL-) 1*β* and IL-6, inflammatory signaling pathways, monolayer permeability of HSAECs, and tumor necrosis factor alpha (ii) Reduction of inflammatory factor expression and nuclear factor-*κ*B activation (iii) Inhibition of activator protein-1, protein kinase C, and mitogen-activated protein kinase expression	[74]

Reduction of overweight and obesity	(i) Change in the composition of intestinal microflora (ii) Restoration of glucose homeostasis (iii) Alleviate insulin resistance	(i) Changes in pathways of sphingolipid and glycerophospholipid metabolisms (ii) Increase of L-carnitine, lysophosphatidylcholine, and sphingomyelin levels (iii) Reduction of phosphatidylcholines	[67]
	(i) Increase in the synthesis of antioxidant enzymes in adipose tissue and in the liver	(i) Increase of expression of antioxidant enzymes and Nrf2 in adipocytes, liver tissue, and HepG2 cells (ii) Regulation of glucose metabolism and liver function	[50]
